# Saúde Mental em Cardiologistas – Uma Preocupação Real

**DOI:** 10.36660/abc.20230028

**Published:** 2023-06-28

**Authors:** Protásio Lemos da Luz

**Affiliations:** 1 Hospital das Clínicas Faculdade de Medicina Universidade de São Paulo São Paulo SP Brasil Instituto do Coração do Hospital das Clínicas da Faculdade de Medicina da Universidade de São Paulo, São Paulo, SP – Brasil

**Keywords:** Transtornos mentais, Suicídio, Relação Médico-Paciente, Terapia Cognitiva Comportamental, Saúde Mental

Estudo recente^1^ mostrou que 1 em cada 4 cardiologistas sofre de distúrbios emocionais incluindo estresse e outras alterações psiquiátricas. Assédio emocional, discriminação, ser divorciado e idade inferior a 55 anos foram fatores predisponentes. O estudo é internacional e incluiu 5931 cardiologistas entrevistados em 2019, abrangendo todos os continentes. Os respondedores à pesquisa representam apenas 8% dos convidados a participarem dela. A maioria são europeus, brancos, casados e com filhos. Indivíduos em começo de carreira foram os mais afetados. A América Latina contribuiu com 17,7% das respostas. Homens constituíram 77,4% da amostra e mulheres, 22,6%. Portanto, trata-se de amostra seleta que pode não representar todo espectro da população cardiológica. Distúrbios emocionais foram relatados por 28% dos entrevistados. Houve considerável variação regional, mas a América do Sul mostrou o maior índice de distúrbios psicológicos, chegando a 39,3%; já na Ásia observaram-se os índices mais baixos (20,1%). Mulheres foram mais propensas a esses distúrbios, mas também procuraram ajuda psicológica com maior frequência. Esse achado corrobora pesquisa anterior que observou que suicídios são mais frequentes entre mulheres médicas do que na população geral.^2^

Outros pesquisadores já haviam documentado diversos distúrbios emocionais entre médicos, tais como ansiedade, depressão, ideação suicida e suicídios.^3 - 6^ Por exemplo, pesquisas recentes nos EUA documentaram que 42% dos cardiologistas sofrem de “ *burnout* ” (exaustão) e 83% tem algum grau de depressão.^2^ A COVID-19 acentuou muito esses problemas. O fenômeno é universal e parece afetar especialmente as mulheres. Os achados preocupam porque influenciam o desempenho dos médicos e, portanto, impactam diretamente na prática médica.

Várias causas, possivelmente, podem explicar tais achados. Escolha errada da profissão é uma delas; muitos médicos não têm vocação para medicina. A escolha da profissão pode sofrer influências familiares, ambientais ou econômicas e não se basear nas verdadeiras aptidões e inclinações pessoais do candidato. Também contribui o total desconhecimento da natureza da profissão médica pelo jovem aspirante. Por exemplo, lidar com pessoas doentes exige certas características de personalidade que nem todos têm; exige paciência, compreensão humana, desapego, solidariedade, capacidade de doação; enfim, exige amor ao próximo. Mas além do aspecto humano, requer também curiosidade científica, dedicação profunda e contínua ao trabalho e certo desapego aos valores materiais. Medicina não é profissão para enriquecer.

Além disso, há a questão da duração da carreira; com o envelhecimento de todos, a carreira médica se tornou mais longa; quem se formou aos 27 anos, por exemplo, vai trabalhar até os 80 ou mais; são mais de 50 anos. É muito tempo para exercer uma profissão que não lhe dê satisfação. Tudo isso deve ser levado em conta quando se escolhe uma profissão.

Hoje a profissão é assalariada e não mais liberal como antes; portanto os médicos têm que se submeter a regimes de trabalho estabelecidos pelos empregadores, com horários e regras que nem sempre são de seu agrado. Essa circunstância retira do profissional uma variável essencial para a boa qualidade de vida: liberdade para escolher como, onde e quanto trabalhar. O estudo Whitehall,^7^ da Inglaterra, demonstrou quanto a insatisfação no trabalho impacta negativamente na sobrevida e qualidade de vida das pessoas.

Outro ponto crítico é a relação íntima médico/paciente que vem se deteriorando. Com frequência o paciente não tem mais o seu médico e o médico não tem mais o seu paciente; ora, essa relação é a base da confiança que alicerça o exercício da profissão.^8^ Os pacientes se queixam dessa dissociação; mas essa rotura afeta também os médicos. No entanto, atualmente, é a instituição que prevalece! A rotatividade de plantonistas ou de médicos de ambulatório obriga a trocas contínuas. O tempo de atendimento pelo SUS e convênios é exíguo, por volta de 8 minutos.

Nesse período não dá para conhecer uma pessoa; o atendimento médico se torna mecanizado, e o paciente não passa de um número. Porém, a doença não é apenas uma questão física ou bioquímica – ela afeta a pessoa toda, afeta sentimentos, cria insegurança e medo; em suma, afeta a alma da pessoa doente. O médico também sente falta dessa ligação afetuosa com seus pacientes. O respeito e admiração que o paciente sente pelo seu médico evanesce quando esse elo se desfaz.

Por outro lado, os honorários pagos pelos sistemas de saúde são baixos, o que exige que os profissionais vejam muitos pacientes em pouco tempo, contribuindo para o distanciamento das pessoas. O retorno econômico tornou-se exíguo considerando o grande investimento que o estudante tem de fazer para se tornar médico. São 6 anos de graduação mais 3 ou 4 de especialização, com grandes exigências mentais, dedicação e responsabilidades. Embora escolas públicas sejam gratuitas, a concorrência é grande e nem todos conseguem vagas. Enormes gastos financeiros em faculdades privadas são hoje a norma; famílias se endividam para formar um médico e este carrega um fardo para a vida.

Também muitos médicos esperam reconhecimento e gratidão quando ajudaram pessoas doentes oferecendo-lhes ora cura, ora conforto, mas isso nem sempre ocorre; aliás, o mais comum é o esquecimento quando não a pura ingratidão. Além disso, processos judiciais contra médicos se tornaram comuns. Portanto, a aura de bem feitor que circundava o médico no passado hoje está em extinção. O desejo de reconhecimento é inerente ao gênero humano e a falta dele certamente retira um pouco do encanto da profissão.

Por outro lado, a assistência médica pura pode ser rotina cansativa; para quem tem experiência não é mentalmente desafiador. Com frequência os casos são repetitivos, as perguntas dos pacientes são ingênuas demais. Isso cansa. Mesmo relacionamentos entre pessoas normais podem ser difíceis: diferenças de personalidades, culturas, religião, política, empatia ou antipatia, gostos em geral aproximam ou afastam pessoas. Quando as pessoas estão doentes, demandas se acentuam. Particularmente em medicina todo mundo tem opinião. Agora, com “Dr. Google”, os pacientes já chegam no médico com diagnóstico e tratamento. Portanto, é precisa muita paciência! O resultado é: para quem não tem vocação, o exercício da profissão é sacrificado. São muitas horas de trabalho, sem privacidade nem respeito por horários. Já para quem gosta do que faz, a profissão representa oportunidade única de conhecer pessoas, ajudar quem sofre, sentir-se útil. Nada é mais gratificante do que o sorriso agradecido de quem esteve à beira da morte e foi salvo. Essa é a essência da profissão – prestar serviços indispensáveis à vida e à saúde.

Como enfrentar essa crise emocional dos médicos atuais é um desafio considerável. Embora psicólogos e psiquiatras sejam os profissionais mais preparados para indicar abordagens específicas neste cenário, permito-me sugerir algumas medidas gerais, com base em experiências acadêmicas e de prática clínica. Vários caminhos podem ser propostos: A – Fazer o que gosta e para o que se tem aptidão. Hoje os estudantes se qualificam mentalmente muito cedo; aprendem ciências e humanidades rapidamente, mas a maturidade emocional é mais lenta; a escolha da profissão requer maturidade e isso só a vivência proporciona. Talvez os aspirantes a médicos devessem ser expostos, por certo tempo, às realidades da profissão antes de ingressarem nas faculdades. B – Hoje a medicina tem muitas subespecialidades, algumas das quais não envolvem contatos diretos com pacientes nem requerem aceitação da responsabilidade direta na condução de casos; exemplos incluem exames de imagem e inteligência artificial. Atualmente, são de fundamental importância no diagnóstico e encaminhamento de casos. Portanto, existem oportunidades diferentes do cuidado tradicional de pacientes. C – Conviver com jovens é outra maneira eficiente de tornar o exercício profissional estimulante e inspirador. O jovem traz entusiasmo, ideias novas, questionamentos que contribuem para tornar a profissão sempre atrativa. Os mais experientes ensinam e orientam, mas também aprendem e se renovam. Na verdade, é uma maneira de assegurar a ampliação, divulgação e perpetuação de seu trabalho. Compartilhar experiências é uma via para deixar um legado. D – Atualizar-se sempre foi uma necessidade imperiosa na prática médica, mas nunca tão decisiva quanto hoje. A velocidade com que os conhecimentos científicos e as novas tecnologias se desenvolvem não tem paralelo na história humana.^9 , 10^ Portanto, os médicos precisam se atualizar para não se tornarem obsoletos rapidamente. E – Pesquisar/associar-se a grupos de pesquisa pode ser uma maneira inteligente de tornar o exercício profissional excitante; descobrir coisas novas sempre representa motivo de justa satisfação pessoal. F – Terapia Cognitiva Comportamental (TCC)^11^ é a abordagem psicológica mais usada dentro da medicina, porque basicamente trabalha com o presente e capacita os pacientes a desenvolver recursos emocionais adequados para o enfrentamento de distúrbios psicológicos frequentemente associados a problemas médicos orgânicos. Neste cenário, incluem-se perdas concretas e simbólicas bem como mudanças de estilo de vida, alterações comportamentais e ajustes familiares e profissionais. Assim, TCC é um recurso útil, eficaz, que deveria ser usado pelos próprios médicos para preservar sua saúde mental. G – Dividir responsabilidades com outros especialistas é uma necessidade e uma arma para evitar frustrações. Hoje os avanços são muitos, em todas as áreas, e o conhecimento profundo, integral e abrangente tornou-se virtualmente impossível para um só indivíduo. É preciso compartilhar conhecimentos e dividir responsabilidades para o bem de todos. H – Escolas médicas podem contribuir selecionando candidatos pelo perfil de personalidade, interesses, vocação e não apenas pelo conhecimento teórico de disciplinas; entrevista pessoal com o candidato seria valiosa. I – Entidades médicas e sistemas de saúde em geral devem velar pela qualidade emocional de seus médicos adotando regimes de trabalho mais satisfatórios.

Em suma a profissão médica é única, composta de ciência e humanismo, cujo objetivo maior é o bem da humanidade. Desafios, como a preservação da saúde mental dos próprios médicos, devem ser enfrentados, mas não devem nos afastar dos objetivos maiores da nossa profissão. Nossa missão é curar quando possível, mitigar sofrimentos e consolar sempre.
