# Supplementary material for: Saúde Mental em Cardiologistas – Uma Preocupação Real
Source: Arq Bras Cardiol. 2023 Jun 28;120(6):e20230028. [Article in Portuguese] doi: 10.36660/abc.20230028 (PMC10365000; doi:10.36660/abc.20230028)
Supplement: Translation

# Translation: Mental Health in Cardiologists: A Real Concern

**Authors:** Protásio Lemos da Luz

**Affiliations:** 1 Brazil Instituto do Coração do Hospital das Clínicas da Faculdade de Medicina da Universidade de São Paulo, São Paulo, SP – Brazil

**Keywords:** Mental Disorders, Suicide, Physician-Patient Relations, Cognitive Behavioral Therapy, Mental Health

A recent study^1^ has shown that 1 in 4 cardiologists suffer from emotional disturbances, including stress and other psychiatric disorders. Emotional harassment, discrimination, divorce, and age under 55 years were predisposing factors. The international study included 5931 cardiologists interviewed in 2019, covering all continents. Survey respondents represent only 8% of those invited to participate in the survey. The majority were European, White, married, and with children. Individuals at the beginning of their careers were the most affected. Latin America contributed with 17.7% of responses. Men constituted 77.4% of the sample, and women constituted 22.6%. Therefore, it was a select sample that may not represent the entire spectrum of the population of cardiologists. Emotional disturbances were reported by 28% of respondents. There was considerable regional variation, but South America showed the highest rate of psychological disorders, reaching 39.3%; in Asia, the lowest indexes were observed (20.1%). Women were more prone to these disorders, but they also sought psychological help more frequently. This finding corroborates previous research which observed that suicide is more frequent among women doctors than in the general population.^2^

Other researchers had already documented several emotional disorders among physicians, such as anxiety, depression, suicidal ideation, and suicide.^3 - 6^ For example, recent research in the United States has documented that 42% of cardiologists suffer from burnout, and 83% have some degree of depression.^2^ COVID-19 has greatly accentuated these problems. The phenomenon is universal and seems to affect women in particular. These findings are a cause of concern, because they influence the performance of physicians and, therefore, have a direct impact on medical practice.

Multiple causes could possibly explain these findings. Wrong choice of profession is one of them; many doctors do not have a vocation for medicine. The choice of profession may be influenced by family, environment, or economic reasons and may not be based on the candidate’s true aptitudes and personal inclinations. Total lack of knowledge regarding the nature of the medical profession by young candidates. also contributes to this. For example, dealing with people who are ill requires certain personality traits that not everyone possesses; it demands patience, human understanding, detachment, solidarity, and generosity; finally, it requires love of others. Beyond the human aspect, it also requires scientific curiosity, profound and continuous dedication to work, and a certain detachment from material values. Medicine is not a profession for becoming rich.

Furthermore, there is the issue of career duration; with population aging, the medical career lasts longer. Those who graduated at 27, for example, will work until they are 80 or older, for more than 50 years, which is a too long time to pursue a profession that does not provide satisfaction. All this must be taken into account when choosing a profession.

Nowadays, the profession is salaried and no longer liberal as it was in the past; therefore, doctors have to submit to work regimes established by employers, with schedules and rules that are not always to their liking. This circumstance means that professionals lose an essential variable for good quality of life: freedom to choose how, where, and how much to work. The Whitehall study^7^ from England, demonstrated the extent to which dissatisfaction at work negatively impacts people’s survival and quality of life.

Another critical point is the intimate doctor-patient relationship that has been deteriorating. Often patients no longer have their doctors, and doctors no longer have their patients; this relationship is the basis of trust that provides the foundation for exercising the profession.^8^ Patients complain about this dissociation, but this rupture also affects doctors. However, currently, it is the institution that comes first! The rotation of doctors on duty or outpatient doctors requires continuous changes. The consultation time through the Brazilian Unified Health System (SUS) and private health insurance plans is short, approximately 8 minutes.

During such a short period, it is not possible to know a person; medical care becomes mechanized, and the patient becomes nothing more than a number. However, illness is not just a physical or biochemical issue. Illness affects the person as a whole; it influences feelings, creating insecurity and fear. In short, illness affects the person’s soul. Doctors also miss this affectionate connection with their patients. The respect and admiration that patients feel for doctors vanish when that bond is not maintained.

On the other hand, the fees paid by health systems are low, making it necessary for professionals to see many patients in a short time, contributing to distancing between people. The economic return has become insufficient considering the major investment that students must make to become doctors. It takes 6 years to graduate, plus 3 or 4 to complete a specialization, with immense mental demands, dedication, and responsibilities. Although public schools are free, competition is fierce, and not everyone manages to be admitted. Huge financial expenses at private medical school are now the norm. Families become indebted to pay the education of a doctor, who will carry a lifelong burden.

Many doctors also expect recognition and gratitude when they help patients, offering them either healing or comfort, but this is not always the case; in fact, they most commonly deal with forgetfulness, or else pure ingratitude. In addition to this, lawsuits against doctors have become commonplace. Therefore, the beneficent aura that once surrounded doctors is now in extinction. The desire for recognition is inherent to humankind and the lack thereof certainly takes away some of the charm of the profession.

On the other hand, pure medical care can be a tiresome routine; for those with experience, it is not mentally challenging. The cases are often repetitive, and the patients’ questions are too naive. This is tiring. Even normal relationships between people can become strained; differences in personalities, cultures, religion, politics, empathy, aversion, and tastes in general bring people closer or farther apart. When people are sick, demands are accentuated. Particularly in medicine, everyone has an opinion. Nowadays, with “Dr. Google”, patients already come to the doctor with a diagnosis and treatment. Therefore, it takes lots of patience! The result is that, for those who do not have a vocation, the exercise of the profession is burdensome. There are many hours of work, without privacy or respect for schedules. For those who enjoy what they do, the profession represents a unique opportunity to meet people, help those who suffer, and feel useful. Nothing is more rewarding than the grateful smile of someone who was on the verge of death and was saved. This is the essence of the profession: providing services that are indispensable to life and health.

Facing this current emotional crisis is a considerable challenge for doctors. Although psychologists and psychiatrists are the professionals best prepared to indicate specific approaches in this scenario, I would like to suggest some general measures, based on academic experience and clinical practice. The followings paths can be proposed: A – Do what you like and what you have the aptitude for. Today students qualify mentally very early; they learn science and humanities quickly, but emotional maturity is slower; the choice of profession requires maturity, and only experience can provide that. Perhaps aspiring doctors should be exposed to the realities of the profession for a while before entering college. B – Medicine currently has many subspecialties, some of which do not involve direct contact with patients or require accepting direct responsibility for managing cases; examples include imaging and artificial intelligence. Currently, they are of fundamental importance for diagnosis and referral of cases. Thus, there are different opportunities, other than traditional patient care. C – Working with young people is another efficient way to make professional practice stimulating and inspiring. Young people bring enthusiasm, new ideas, and questions that contribute to keeping the profession attractive. Those with more experience teach and guide, but they also learn and renew themselves. In fact, it is a way of ensuring the expansion, dissemination, and perpetuation of their work. Sharing experiences is a way to leave a legacy. D – Staying up to date has always been an imperative need in medical practice, but it has never been as decisive as it is today. The speed with which scientific knowledge and new technologies are developing is unparalleled in human history.^9 , 10^ Therefore, doctors need to remain up to date in order not to become rapidly obsolete. E – Researching/joining research groups can be an intelligent way to make professional practice exciting; discovering new things always provides a reason for well-deserved personal satisfaction. F – Cognitive Behavioral Therapy^11^ is the most widely used psychological approach within medicine, because it basically works with the present and enables patients to develop adequate emotional resources for coping with psychological disorders often associated with organic medical problems. In this scenario, concrete and symbolic losses are included, as well as lifestyle changes, behavioral changes, and family and professional adjustments. Thus, Cognitive Behavioral Therapy is a useful, effective resource that should be used by doctors themselves to preserve their mental health. G – Sharing responsibilities with other specialists is a necessity and a means to avoid frustration. There are currently many advances, in all areas, and profound, integral and comprehensive knowledge has become virtually impossible for a sole individual. It is necessary to share knowledge and responsibilities for the good of all. H – Medical schools can contribute by selecting candidates based on their personality profile, interests, vocation and not merely on theoretical knowledge of disciplines; personal interviews with candidates would be valuable. I – Medical entities and health systems in general must ensure the emotional quality of doctors by adopting more satisfactory work regimes.

In conclusion, the medical profession is unique, composed of science and humanism, and its main objective is the good of humanity. It is necessary to face challenges, such as preserving the mental health of doctors, but these challenges must not lead us away from the greater goals of our profession. Our mission is to heal whenever possible, to mitigate suffering, and always to console.
